# Radiofluorination of an Anionic, Azide-Functionalized Teroligomer by Copper-Catalyzed Azide-Alkyne Cycloaddition

**DOI:** 10.3390/nano13142095

**Published:** 2023-07-18

**Authors:** Barbara Wenzel, Maximilian Schmid, Rodrigo Teodoro, Rareş-Petru Moldovan, Thu Hang Lai, Franziska Mitrach, Klaus Kopka, Björn Fischer, Michaela Schulz-Siegmund, Peter Brust, Michael C. Hacker

**Affiliations:** 1 Department of Neuroradiopharmaceuticals, Institute of Radiopharmaceutical Cancer Research, Helmholtz-Zentrum Dresden-Rossendorf, 04318 Leipzig, Germany; r.teodoro@life-mi.com (R.T.); r.moldovan@hzdr.de (R.-P.M.); t.lai@hzdr.de (T.H.L.); k.kopka@hzdr.de (K.K.); peterbrustdeu@aol.com (P.B.); 2 Institute of Pharmacy, Pharmaceutical Technology, Leipzig University, 04317 Leipzig, Germany; maximilian.schmid@uni-leipzig.de (M.S.); franziska.mitrach@uni-leipzig.de (F.M.); schulz@uni-leipzig.de (M.S.-S.); 3 Institute of Pharmaceutics and Biopharmaceutics, Heinrich Heine University Düsseldorf, 40225 Düsseldorf, Germany; bjoern.fischer@hhu.de; 4 Faculty of Chemistry and Food Chemistry, School of Science, Technical University Dresden, 01069 Dresden, Germany

**Keywords:** teroligomer, fluorine-18, ^18^F-polymer, click reaction, CuAAC, PEG-[^18^F]FPyKYNE

## Abstract

This study describes the synthesis, radiofluorination and purification of an anionic amphiphilic teroligomer developed as a stabilizer for siRNA-loaded calcium phosphate nanoparticles (CaP-NPs). As the stabilizing amphiphile accumulates on nanoparticle surfaces, the fluorine-18-labeled polymer should enable to track the distribution of the CaP-NPs in brain tumors by positron emission tomography after application by convection-enhanced delivery. At first, an unmodified teroligomer was synthesized with a number average molecular weight of 4550 ± 20 Da by free radical polymerization of a defined composition of methoxy-PEG-monomethacrylate, tetradecyl acrylate and maleic anhydride. Subsequent derivatization of anhydrides with azido-TEG-amine provided an azido-functionalized polymer precursor (**o14PEGMA-N_3_**) for radiofluorination. The ^18^F-labeling was accomplished through the copper-catalyzed cycloaddition of **o14PEGMA-N_3_** with diethylene glycol–alkyne-substituted heteroaromatic prosthetic group **[^18^F]2,** which was synthesized with a radiochemical yield (RCY) of about 38% within 60 min using a radiosynthesis module. The ^18^F-labeled polymer **[^18^F]fluoro-o14PEGMA** was obtained after a short reaction time of 2–3 min by using CuSO_4_/sodium ascorbate at 90 °C. Purification was performed by solid-phase extraction on an anion-exchange cartridge followed by size-exclusion chromatography to obtain **[^18^F]fluoro-o14PEGMA** with a high radiochemical purity and an RCY of about 15%.

## 1. Introduction

Amphiphilic polymers and nanomaterials have emerged as promising platforms for cancer therapy due to their unique properties, such as tunable size and shape, high surface-area-to-volume ratio and ability to target tumor cells. Therapeutic effects are associated with the observation that larger molecular systems can passively accumulate in tumors due to the *enhanced permeability and retention* (*EPR*) effect first described by Maeda et al. [1]. Synthetic polymers of the organic type can be effectively controlled in their composition and appearance and offer the advantage of convenient structural modifications. This motivates the intense investigation into polymeric nanosystems for the targeted delivery of drugs or imaging probes [2,3,4,5,6,7].

The topic of this research collaboration is the development of calcium phosphate nanoparticles (CaP-NPs) stabilized by suitable polymers and loaded with small interfering RNA (siRNA) in order to investigate their potential for a targeted localized tumor therapy via convection-enhanced delivery (CED). CED is a technique to deliver therapeutics directly to the tumor using one or more stereotactically placed catheters [8]. For the pharmacotherapy of brain tumors this method bears particular advantages, such as bypassing the blood–brain barrier with consequently increasing drug doses at the therapeutic side and less systemic side effects compared to conventional application forms. In a first step, we have recently developed CaP-NPs stabilized with polymeric amphiphiles as a suitable siRNA carrier material [9]. One future intention of this research project is to investigate the distribution of these loaded CaP-NPs after delivery to the desired brain region in rats using positron emission tomography (PET). PET is a non-invasive imaging technique that allows the visualization of the distribution of radioactive substances in vitro and in vivo. The PET radionuclide fluorine-18 was selected for our purpose because of its reasonable half-life (109.7 min) and chemical properties that allow for covalent incorporation in molecules, which often ensures a higher stability of the radiolabel as compared to complexed radiometals. Therefore, the development and synthesis of an ^18^F-radiolabeled polymer was needed as it fulfilled the requirements with respect to stabilizing as well as imaging of the nanoparticles.

To date, only a few ^18^F-labeled polymers have been described in the literature. In 2009, Herth et al. [10] reported on the synthesis of *N*-(2-hydroxypropyl)-methylacrylamide (HPMA)-based polymeric structures, which were radiolabeled by coupling the phenolic tyramine functionalities of the polymer with 2-[^18^F]fluoroethyl-1-tosylate ([^18^F]FETos). This procedure was used for development and preclinical PET studies of HPMA-based polymeric conjugates [10,11,12]. In an approach to generate ^18^F-labeled polyester-based nanoparticles, Di Mauro and co-workers [13] used 4-[^18^F]fluorobenzyl-2-bromoacetamide ([^18^F]FBBA) for condensation with a thiol-functionalized polyethylenglycolic ester and subsequently prepared nanoparticles from the radiofluorinated polymer. Highly efficient cycloaddition reactions, so called click reactions, which are well established for ^18^F-labeling of sensitive biomolecules, have also been used for the generation of radiolabeled polymers by instant conjugation to functionalized polymers. As one example, the copper-catalyzed azide-alkyne cycloaddition (CuAAC) of 1-azido-2-(2-(2-[^18^F]-fluoroethoxy)ethoxy)ethane to different alkyne-functionalized polymers has been described [14,15]. Moreover, one example for a copper-free strain-promoted azide-alkyne cycloaddition (SPAAC) of [^18^F]fluoroethylazide with a cyclooctyne-functionalized hydrophilic polymer (ethyl poly(2-ethyl-2-oxazoline)) was published [16]. 

**Scheme 1:**
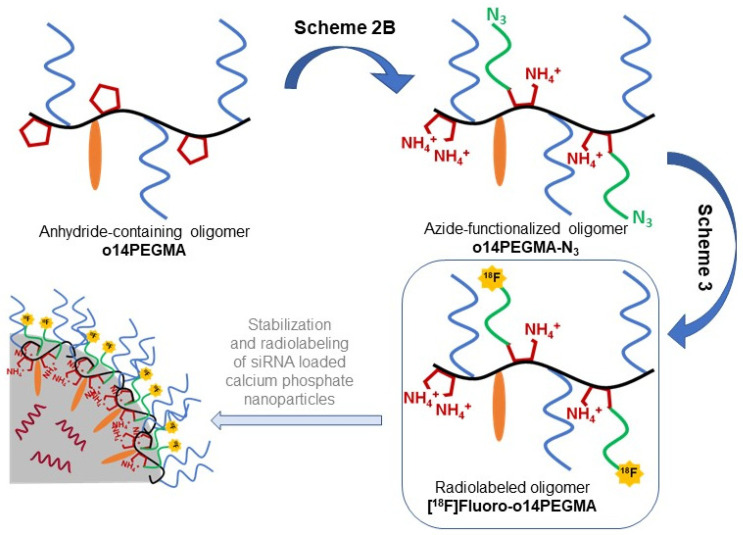
Schematic illustration of the material concept investigated in this study. An amphiphilic anhydride group-containing oligomer (**o14PEGMA**) is synthesized from tetradecyl acrylate (orange), mPEG-MAc (blue) and maleic anhydride (red) [9]. The anhydride groups are derivatized, and unreacted anhydrides are hydrolyzed, yielding the azide-functionalized (green) teroligomer **o14PEGMA-N_3_** (see also Scheme 2B). The water-soluble oligomer is then conjugated with a suitable alkyne-bearing and radiolabeled group in a copper-catalyzed azide-alkyne cycloaddition, yielding **[^18^F]fluoro-o14PEGMA** (see also Scheme 3). The radiofluorinated oligomer would then be available as a stabilizer for the fabrication of siRNA-loaded CaP-NPs in analogy to our previous study [9].

Copper-catalyzed click reactions are known for their mild conditions, the use of aqueous reaction media and a fast conversion rate. In particular, the latter is of importance for our purpose due to the relatively short half-life of the fluorine-18 radionuclide, which limits the synthesis time. We therefore focused on this reaction type, in which the polymer provides the azide functionalities for coupling with a suitable alkyne bearing and radiolabeled group. Because of its relatively hydrophilic character suitable for the desired reaction conditions, [^18^F]2-fluoro-3-(2-(2-(prop-2-ynyloxy)ethoxy)ethoxy)pyridine (**[^18^F]2**, in the literature is named as PEG-[^18^F]FPyKYNE) [17,18] was selected as the alkyne-bearing group. The concept of the entire study is illustrated in Scheme 1. 

Here, we describe the two-step ^18^F-radiolabeling procedure and purification of an anionic amphiphilic teroligomer accomplished by the copper-catalyzed cycloaddition of the alkyne-substituted aromatic group **[^18^F]2** to an azide-functionalized amphiphilic oligomer. 

## 2. Materials and Methods

### 2.1. Oligomer Synthesis

#### 2.1.1. Materials

Tetrahydrofuran (THF) and diethyl ether were HPLC-grade and obtained from VWR International GmbH (Darmstadt, Germany). For polymer synthesis, THF was dried by refluxing over potassium and sodium and subsequently distilled. Maleic anhydride (MA) and aniline were purchased from Thermo Fisher Scientific and VWR International GmbH, respectively. Tetradecyl acrylate (TDA) and poly(ethylene glycol) methyl ether methacrylate (methoxy-PEG-monomethacrylate, mPEG-MAc) with an average Mn of 950 were obtained from TCI Deutschland GmbH (Eschborn, Germany) and used as received. 2,2′-Azobis(2-methylpropionitril) (AIBN), 2-(2-(2-(2-azidoethoxy)ethoxy)ethoxy)ethan-1-amine (azido-TEG-amine) and triethylamine (TEA) were from Sigma-Aldrich Chemie GmbH (Taufkirchen, Germany). Acetone was purchased from Carl Roth GmbH + Co. KG (Karlsruhe, Germany), and aqueous ammonia (25% m/V) was obtained from Grüssing GmbH (Filsum, Germany). Deuterated solvents, CDCl_3_, DMSO-D6, both with tetramethyl silane, and D_2_O were purchased from ARMAR GmbH (Leipzig, Germany). Float-A-Lyzer^®^ dialysis devices from Repligen Europe B.V. (Dreda, The Netherlands), with a cellulose ester membrane and a molecular weight cut off of 0.1–0.5 kDa were used.

#### 2.1.2. Synthesis of Pristine Teroligomer **o14PEGMA** (**1:1:2.5**)

The synthesis of the pristine teroligomer oligo(TDA-co-mPEG-MAc-co-MA) (**o14PEGMA**) composed of TDA (14 in oligomer code), mPEG-MAc (PEG in oligomer code) and MA was performed as recently described [9]. In brief, the three comonomers were mixed in a molar ratio of 1:1:2.5 (TDA/mPEG-MAc/MA) in quantities of 4.1 mL (13.3 mmol) of TDA, 11.5 mL (13.3 mmol) of mPEG-MAc and 3.27 g (33.3 mmol) of MA. The comonomers were dissolved or diluted in an appropriate amount of THF and added to the flask at 60 °C. The final reaction volume was 300 mL (total comonomer concentration: 0.2 mol/L). After addition of 0.2 g of AIBN, the reaction mixture was stirred for 18 h and concentrated by rotary evaporation. The crude product was three times precipitated in diethyl ether, isolated and vacuum dried over several days. The resulting purified **o14PEGMA** was analyzed by gel permeation chromatography (GPC) and ^1^H NMR as described before [9]. The amount of incorporated anhydride groups and the chemical intactness were determined before and after hydrolysis using conductometric titration and by a titration method according to Brown and Fujimorij after anhydride aminolysis [19]. 

#### 2.1.3. Synthesis of Azide-Modified Teroligomer and Isolation as Ammonium Salt (**o14PEGMA-N_3_**)

Based on the results of the anhydride quantification by titration and molecular size determination by GPC, the pristine teroligomer **o14PEGMA** was derivatized with azido-TEG-amine in a molar ratio that was set to 25% of intact anhydrides (8.4 μmol). TEA was added in an amount equal to 5% of chemically intact anhydrides. In a typical batch, azido-TEG-amine (0.471 µL, 2.1 μmol) and base (0.060 µL, 427 nmol) were added to 100 mg (22 μmol) of **o14PEGMA** dissolved in acetone and magnetically stirred at room temperature for 4 h. After removal of the volatile components by vacuum drying, the raw product was dissolved in aqueous ammonia (1 M) and maintained at 40 °C for 12 h [20] to hydrolyze any remaining anhydride and to form the ammonium salt **o14PEGMA-N_3_**. Then, the reaction mixture was concentrated by rotary evaporation and subjected to dialysis against water for 10 h with four changes of the outer phase to obtain purified **o14PEGMA-N_3_** as a sticky white hygroscopic solid. Proton NMR analysis of the linker modified oligomer **o14PEGMA-N_3_** was inconclusive, because it was impossible to distinguish between TEG-azide and the mPEG-MAc chains, which are chemically identical, an observation which has already been described in the literature [21]. 

#### 2.1.4. Fourier Transform Infrared Spectroscopy (FT-IR) and Confocal Raman Spectroscopy

FT-IR and confocal Raman spectroscopy were applied to visualize the amidation of **o14PEGMA** with azido-TEG-amine. FT-IR analysis of samples was performed on a Nicolet iS™ 50-FT-IR with a Smart Performer Sample Unit (Thermo Scientific) equipped with the Omnic Spectra Software 2.2.4.3 provided with the instrument. 

Samples of the dry polymer were also investigated using the confocal Raman microscope alpha-300 R (WITec, Ulm, Germany). A single mode laser with a wavelength of 532 nm was applied for excitation. Using a Zeiss EC Epiplan-Neofluar Dic 50x/0.8 microscope objective, the laser power on the samples was set to 20 mW. The Raman microscope was equipped with a WITec UHTS 300 spectrometer and an Andor iDus Deep Depletion CCD camera, which was cooled down to −60 °C. By using a reflection grating with 600 lines/mm, an average spectral resolution of 3.8 cm^−1^/pixel was achieved. Raman spectra were recorded using an exposure time of 20 s by accumulating 10 × 2 s. For the data interpretation, WITec FIVE 5.3.18.110 software was used. Samples were randomly measured at three positions, spectra were merged, baseline-corrected and -normalized.

### 2.2. Radiochemistry

#### 2.2.1. Synthesis of Non-Radioactive Reference and Precursor

The compound 2-fluoro-3-(2-(2-(prop-2-ynyloxy)ethoxy)ethoxy)pyridine **2** and the corresponding trimethyl ammonium trifluoromethanesulfonate precursor **1** were synthesized as reported [17]. The identity of the compounds was controlled by NMR (spectra in Figure S1 in Supplementary Materials).

[3-(2-(Prop-2-ynyloxy)ethoxy)ethoxy)pyridine-2yl] trimethylammonium trifluoromethanesulfonate (**1**): ^1^H NMR (300 MHz, CDCl_3_) *δ*: 7.85 (dd, *J* = 4.9, 1.5 Hz, 1H), 7.01 (dd, *J* = 7.8, 1.5 Hz, 1H), 6.71 (dd, *J* = 7.8, 4.9 Hz, 1H), 4.21 (t, *J* = 2.2 Hz, 2H), 4.13 (dd, *J* = 5.6, 4.2 Hz, 2H), 3.89 (dd, *J* = 5.6, 4.3 Hz, 2H), 3.82–3.55 (m, 4H), 3.00 (s, 9H), 2.42 (t, *J* = 2.4 Hz, 1H).

2-Fluoro-3-(2-(2-(prop-2-yn-1-yloxy)ethoxy)ethoxy)pyridine (**2**): ^1^H NMR (400 MHz, CDCl_3_) *δ*: 7.75 (dt, *J* = 4.8, 1.6 Hz, 1H), 7.42–7.28 (m, 1H), 7.10 (ddd, *J* = 7.9, 4.9, 0.6 Hz, 1H), 4.41–4.12 (m, 4H), 4.01–3.84 (m, 2H), 3.83–3.73 (m, 2H), 3.73–3.57 (m, 2H), 2.44 (t, *J* = 2.4 Hz, 1H).

#### 2.2.2. Analytics

Radio-thin-layer chromatography (radio-TLC) of the prosthetic group **[^18^F]2** was performed on plates pre-coated with silica gel (Polygram^®^ SIL G/UV254) and with ethyl acetate/*n*-hexane (3:1, *v*/*v*) as eluent. The plates were exposed to storage phosphor screens (BAS IP MS 2025 E, GE Healthcare Europe GmbH, Freiburg, Germany) and recorded using the Amersham Typhoon RGB Biomolecular Imager (GE Healthcare Life Sciences). Images were quantified using ImageQuant TL8.1 software (GE Healthcare Life Sciences). 

Analytical radio-HPLC separations were performed on either (i) a JASCO LC-2000 system, incorporating a PU-2080Plus pump, AS-2055Plus auto injector (100 µL sample loop), and a UV-2070Plus (JASCO Deutschland GmbH, Pfungstadt, Germany) detector coupled with a radioactivity HPLC flow monitor (Gabi Star, raytest Isotopenmessgeräte GmbH, Straubenhardt, Germany) or (ii) a JASCO LC-4000 system, incorporating a PU-4180-LPG pump, AS-4050 auto injector (100 µL sample loop) and a UV-diode array detector MD-4015 coupled with a radio flow monitor (Gabi Nova, Elysia-raytest GmbH, Straubenhardt, Germany). Data analysis was performed either using Galaxy chromatography (Agilent Technologies) or ChromNAV 2.3C (JASCO Deutschland GmbH, Pfungstadt, Germany) software. For **[^18^F]2,** a Reprosil-Pur C18-AQ column (250 × 4.6 mm; 5 µm; Dr. Maisch HPLC GmbH; Ammerbuch-Entringen, Germany) with ACN/aq. 20 mM NH_4_OAc (pH 6.8) as eluent mixture and a flow of 1.0 mL/min was used (gradient: eluent A 10% ACN/aq. 20 mM NH_4_OAc; eluent B 90% ACN/aq. 20 mM NH_4_OAc; 0–5 min 100% A, 5–25 min up to 100% B, 25–29 min 100% B, 29–30 min up to 100% A, 30–35 min 100% A). For the radiolabeled polymer **[^18^F]o14PEGMA** the following systems were used: (1) A HiTrap^TM^ Desalting 5 mL column (GE Healthcare Europe GmbH, Freiburg, Germany) with ethanol/aq. 25 mM sodium phosphate buffer (pH 7.0) as eluent mixture and a flow of 1.0 mL/min (either in gradient mode with eluent A of 100% ethanol and eluent B of 100% aq. 25 mM sodium phosphate pH 7; 0–10 min 10% A, 10–11 min up to 25% A, 11–17 min 25% A, 17–18 min up to 10% A, 18–25 min 10% A; or in isocratic mode with 10% A and 90% B) and (2) a Reprosil-Pur C18-AQ column (250 × 4.6 mm; 5 µm; Dr. Maisch HPLC GmbH; Ammerbuch-Entringen, Germany) with ACN/aq. 20 mM NH_4_OAc (pH 6.8) as eluent mixture and a flow of 1.0 mL/min (gradient: eluent A 10% ACN/aq. 20 mM NH_4_OAc; eluent B 90% ACN/aq. 20 mM NH_4_OAc; 0–10 min 100% A, 10–25 min up to 100% B, 25–30 min 100% B, 30–31 min up to 100% A, 31–35 min 100% A). 

The ammonium acetate and sodium phosphate concentration, stated as aq. 20 mM NH_4_OAc and aq. 25 mM sodium phosphate, respectively, corresponds to the concentration in the aqueous component of an eluent mixture.

#### 2.2.3. Radiosynthesis of **[^18^F]2**

Remotely controlled radiosynthesis of **[^18^F]2** (formerly named PEG-[^18^F]PyKYNE) [17] was performed using a TRACERlab FX2 N synthesis module (GE Healthcare, Chicago, IL, USA) equipped with a Laboport vacuum pump N810.3FT.18 (KNF Neuburger GmbH, Freiburg, Germany), a BlueShadow UV detector 10D (KNAUER GmbH, Berlin, Germany) and TRACERlab FX software. No-carrier-added [^18^F]fluoride was produced via the [^18^O(p,n)^18^F] nuclear reaction by irradiation of an [^18^O]H_2_O target (Hyox 18 enriched water, Rotem Industries Ltd., Mishor Yamin D.N AravaCity, Israel) on a Cyclone 18/9 (iba RadioPharma Solutions, Louvain-la-Neuve, Belgium) with a fixed energy proton beam using a Nirta [^18^F]fluoride XL target. 

**Figure 1:**
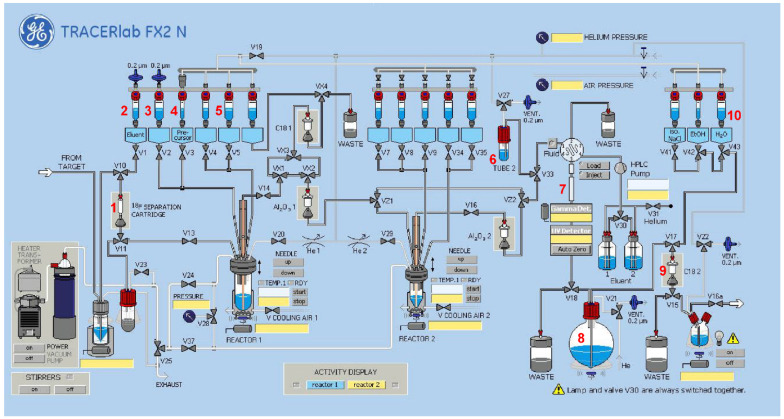
Flow sheet of the synthesis module TRACERlab FX2 N for the radiosynthesis of **[^18^F]2**. (1) Sep-Pak Accell Plus QMA Carbonate Plus light cartridge, (2) K_2_CO_3_ (1.8 mg (13 µmol) in 400 µL water, 100 µL ACN), (3) K_2.2.2._ (11 mg (29 mmol) in 1.5 mL of ACN), (4) precursor **1** (1.0–1.5 mg (2.3–3.5 µmol) in 800 µL DMSO), (5) 3.5 mL of water + 0.5 mL of ACN, (6) injection vial, (7) Reprosil-Pur C18-AQ, 250 × 10 mm (ACN/H_2_O/TFA (35:75:0.05, *v*/*v*/*v*), flow 4.0 mL/min), (8) 50 mL of water + 40 µL of 1M aq. NaOH, (9) Sep-Pak^®^ C18 light, (10) 2 mL of water.

As illustrated in the flow sheet of the synthesis module (Figure 1), [^18^F]fluoride (4–6 GBq) was trapped on a Sep-Pak Accell Plus QMA Carbonate Plus light cartridge (Figure 1, entry 1; Waters GmbH, Eschborn, Germany) and eluted into the reactor with potassium carbonate (K_2_CO_3_, 1.8 mg, 13 µmol; entry 2) dissolved in 400 µL of water and 100 µL of ACN. After the addition of Kryptofix 2.2.2. in 1.5 mL ACN (11 mg, 29 µmol, entry 3), the mixture was dried by azeotropic distillation for 5 min at 65 °C and for 2 min at 85 °C. Thereafter, 1.0–1.5 mg of the trimethylammonium triflate precursor **1** dissolved in 800 µL of DMSO (entry 4) was added, and the reaction mixture was stirred at 120 °C for 8 min. After cooling, the reaction mixture was diluted with 3.5 mL of water and 0.5 mL of ACN and transferred into the injection vial (entry 6). Semi-preparative HPLC was performed using a Reprosil-Pur C18-AQ column (entry 7, 250 × 10 mm, Dr. Maisch HPLC GmbH, Ammerbuch-Entringen, Germany) with ACN/H_2_O/TFA (35:75:0.05, *v*/*v*/*v*) as eluent at a flow of 4.5 mL/min. **[^18^F]2** was collected into the dilution vessel (entry 8) previously loaded with 50 mL H_2_O and 40 µL 1M aq. NaOH at retention times of 15–17 min. Final purification was performed by passing the solution through a Sep-Pak^®^ C18 light cartridge (entry 9; Waters GmbH, Eschborn, Germany), followed by washing with 2 mL of water (entry 10). The cartridge was then removed from the automat and the trapped prosthetic group was manually eluted with 300–400 µL DMSO to prepare it for the subsequent click reaction. The quality control of the product was performed using radio-TLC and radio-HPLC.

#### 2.2.4. Radiosynthesis of the Teroligomer **[^18^F]fluoro-o14PEGMA** by Click-Conjugation of **[^18^F]2**

The CuAAC reactions of **[^18^F]2** with azido-TEG-amine were initially performed to identify suitable reaction parameters and are described in the Supplementary Material.

For the generation of the radiolabeled polymer **[^18^F]fluoro-o14PEGMA** a typical radiosynthesis procedure was performed as follows: The azide functionalized teroligomer **o14PEGMA-N_3_** (2.0 mg, 0.44 µmol) was dissolved in 310 µL of water and mixed with 72 µL (36 µmol) of a freshly prepared aqueous 0.5 M sodium ascorbate solution and **[^18^F]2** (150–350 MBq) in 100 µL of DMSO. Thereafter, 18 µL (9 µmol) of an aqueous 0.5 M CuSO_4_ solution was added under stirring, and the reaction mixture was heated up to 90 °C for 2–3 min. The reaction was performed under argon atmosphere, and all solvents were saturated with argon before usage. For the next step, the solution was cooled on ice, diluted with 20 mL of water and loaded on a Chromafix SB cartridge (size M, Macherey-Nagel GmbH & Co., KG, Düren, Germany), which was preconditioned with 10 mL of ethanol and 10 mL of water. The loaded cartridge was washed with 3 mL of water, and the product eluted with 1.7 mL of an aq. 1.0 M HCl solution. The obtained eluate was neutralized with about 0.3 mL of an aq. 5.0 M NaOH solution and directly injected in the semi-preparative size-exclusion chromatography (SEC) system (JASCO LC-2000 module with a PU-2080-20 pump and an UV/VIS-2075 detector; a radioactivity HPLC detector, whose measurement geometry was slightly modified (Gabi Star, Elysia-raytest GmbH); and a fraction collector (Advantec CHF-122SC)). Two in-line connected HiTrap^TM^ Desalting 5 mL columns (GE Healthcare Europe GmbH, Freiburg, Germany) were used with aq. 3.75 mM sodium phosphate buffer (pH 7) as eluent and a flow of 1.0 mL/min.

## 3. Results and Discussion

### 3.1. Polymer Synthesis and Characterization

**Scheme 2:**
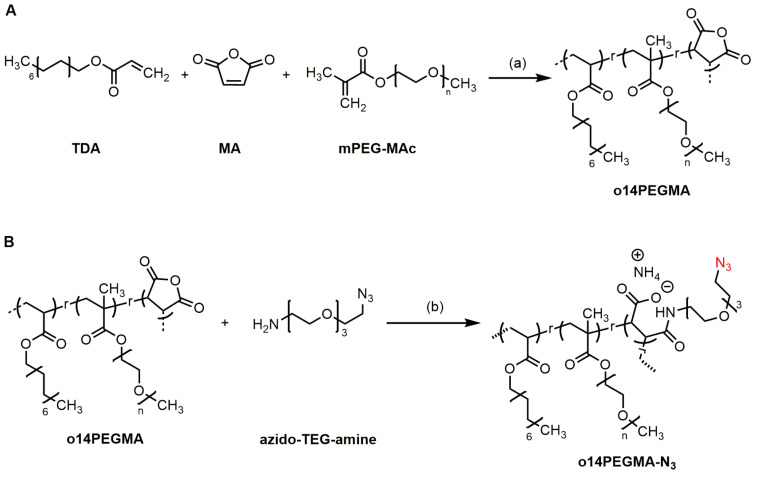
Synthesis of (**A**) the pristine teroligomer **o14PEGMA** and (**B**) the azide-functionalized teroligomer **o14PEGMA-N_3_**. Reagents and conditions: (a) free-radical polymerization with AIBN, 60 °C for 18 h under magnetic stirring; (b) (i) triethylamine in acetone, stirring at RT for 4 h, (ii) removal of acetone under vacuum; (iii) 1M aqueous ammonia, magnetic stirring at 40 °C for 12 h.

**Scheme 3:**
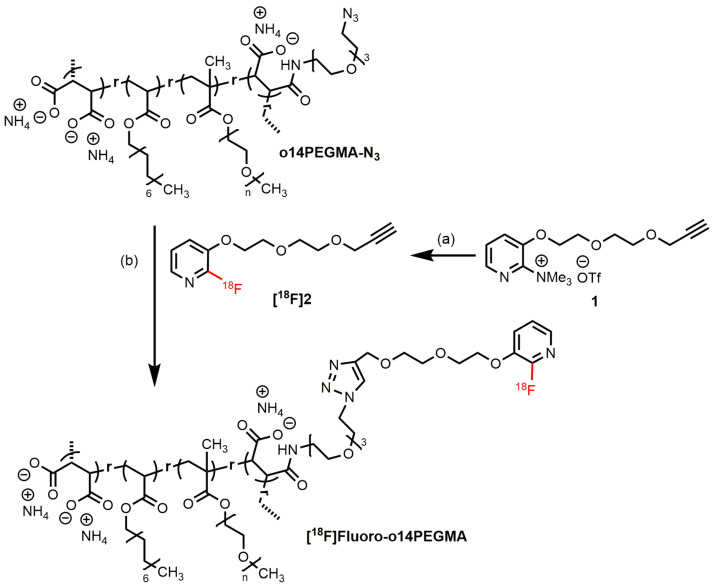
Radiosynthesis of the teroligomer **[^18^F]fluoro-o14PEGMA** by copper-catalyzed azide-alkyne cycloaddition of **o14PEGMA-N_3_** and **[^18^F]2**. Reaction conditions: (a) [^18^F]F^-^/K_222_/K_2_CO_3_, DMSO, 120 °C, 8 min; (b) NaAsc/CuSO_4_ 4:1, DMSO/H_2_O 1:4, 90 °C, 2–3 min.

With the aim to synthesize an ^18^F-labeled amphiphilic polymer suitable for the generation of siRNA-loaded calcium phosphate nanoparticles as potential therapeutics for a targeted localized tumor application via CED, the azide-functionalized anionic teroligomer **o14PEGMA-N_3_** was developed as precursor for a two-step ^18^F-radiolabeling strategy, as depicted in Scheme 3. As a basis for the functionalization, the anhydride-containing teroligomer **o14PEGMA** was synthesized at first following a previously in our consortium developed free radical polymerization protocol (Scheme 2A) [9,22,23]. The molar feed used during the synthesis correlated to a mass percentage of the three building blocks of 18% TDA, 17% MA and 65% mPEG-MAc. TDA, a fatty alcohol acrylate derivative with a saturated chain, was chosen as hydrophobic component. A medium chain length was selected with regard to the respective membrane interaction and cellular uptake of the intended teroligomer-modified nanoparticles. Similar chain length can be found in phospholipids, such as 1,2-dimyristoyl-*sn*-glycero-3-phosphocholine, which has frequently been used for nanoscale drug delivery [24] or slightly altered as 1-monoethoxypolyethyleneglycol-2,3-dimyristylglycerol in the mRNA 1273 vaccine [25]. These examples indicate that the myristyl residue is compliant with the cellular uptake of nanostructures.

Another key factor is the amount of intact anhydrides, as their integrity influences the efficiency of modification and ionic interactions as well as exhibiting an appropriate balance to stabilize nanoparticles, as is currently reported [9]. The combination of two different titration methods revealed an anhydride intactness of 82%, which is in an expected range in comparison to similar anhydride-containing oligomers [23,26,27].

PEG is well known and widely used to prolong circulation time of submicron drug-delivery vehicles in the bloodstream and to reduce opsonization by immune cells [28,29]. However, it is important to balance the PEG content of the teroligomer. While PEG structures contribute to physical stabilization of the nanoparticles and prevent their aggregation, high contents should be avoided because they can reduce cellular uptake, binding efficiency and therefore the final efficacy of the therapeutic approach [30].

The number average molecular weight of the teroligomer was obtained by GPC as 4550 ± 20 Da (Đ_M_ = 1.71 ± 0.0) [9]. The molecular weight is small enough to ensure for renal elimination but, at the same time, appropriate for interaction with a nanoparticle surface [31].

### 3.2. Linker Modification

In order to render the teroligomer accessible for ^18^F-radiolabeling with the prosthetic group **[^18^F]2** via copper-catalyzed click reaction, **o14PEGMA** was derivatized with azido-TEG-amine and isolated as the corresponding ammonium salt (**o14PEGMA-N_3_**, Scheme 2B). The composition was determined by combining ^1^H NMR results (mPEG-MAc and TDA content) with the MA content from the conductometric titration method. Based on these results, it can be concluded that the azido-TEG-amine linker group was successfully implemented into the teroligomer structure.

**Figure 2:**
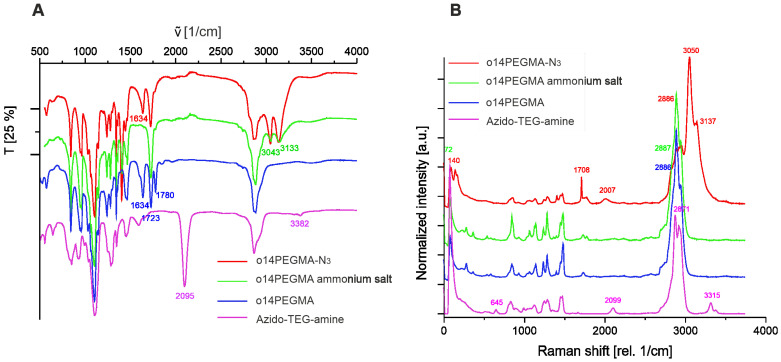
Spectra of **o14PEGMA-N_3_**, **o14PEGMA**, the ammonium salt of **o14PEGMA** and azido-TEG-amine obtained by (**A**) FT-IR and (**B**) confocal Raman spectroscopy. Both methods complementarily indicate that azido-TEG-amine could be grafted on the **o14PEGMA** backbone by aminolysis. Additional peaks originating from azido-TEG-amine are visible in the reaction product **o14PEGMA-N_3_** when compared to the non-derivatized ammonium salt of **o14PEGMA**.

Further structural characterization of **o14PEGMA-N_3_** was performed by FT-IR and confocal Raman spectroscopy. As shown in Figure 2A, the FT-IR spectra of azido-TEG-amine displayed a prominent peak at 2095 cm^−1^, representing the asymmetric stretch modes of the delocalized azide–nitrogen double bond [32]. This signal of the azide group could not be clearly detected after attachment of the linker to the teroligomer and subsequent aminolysis. Nevertheless, we expect that a covalent derivatization was achieved, but the number of azide groups in the derivatized oligomer was below the detection limit of the FT-IR equipment. Despite the low number of azide groups, the ^18^F-labeling could be achieved as will be shown in subsequent paragraphs. Beside the low concentration of the azido group, there is also a possibility that the signal has been shifted due to hydrogen bonding or interactions with the ammonium cations. Furthermore, the automatic atmospheric vapor compensation that affects this area of the spectrum could also have affected any small signal derived from the azide group. Nevertheless, an amide I symmetric stretch vibration of the amide carbonyl peak is clearly visible at 1635 cm^−1^ in the azide-modified oligomer **o14PEGMA-N_3_**, which indicates a successful amidation with the linker molecule [33]. Clear evidence of ammonium salt formation can also be seen in the 3000 cm^−1^ region of the spectrum. At 1402 cm^−1^, the amide III region is represented as an overlap of the symmetric stretch along the carbon nitrogen bond and the nitrogen hydrogen bond deformation in phase vibrations. Due to its deformation origin from the nitrogen hydrogen bond, this band also occurs in the ammonium salt of the unmodified oligomer [34]. At 1718 cm^−1^, the symmetric stretching of the strong carbonyl bond originates from the acid form of the maleic anhydride. It is notable that, as described in the literature, there is a shoulder in the anhydride position at around 1780 cm^−1^ indicating that some chemically intact anhydrides can be found after hydrolysis due to chemical equilibrium [35]. As expected, the highest number of chemically intact anhydrides are found in the pristine oligomer. The band at 1637 cm^−1^ is indicative of carboxylate OH bending during interactions with bound water [36]. Taken together, indications of covalent attachment of the azide linker molecules to the oligomers have been seen with FT-IR. The analysis also shows that the anhydride moieties of the teroligomers were effectively hydrolyzed and transformed to their corresponding ammonium salts. 

Spectra recorded by confocal Raman spectroscopy (Figure 2B) also provided indications for successful azide modification. The azide group reveals signals at 645, 2099 and 3315 cm^−1^, whereas a Raman transition was found around 2007 cm^−1^ for the azide-derivatized oligomer **o14PEGMA-N_3_** [37]. The observation confirms interactions of the azide group with other functional moieties of the oligomer, which has already been suspected from the FT-IR data.

### 3.3. Radiochemistry

#### 3.3.1. Radiosynthesis of the Alkyne-Substituted Heteroaromatic Group **[^18^F]2**

Radiofluorination of **[^18^F]2**, needed for coupling with the azide-modified oligomer **o14PEGMA-N_3_,** was achieved by a nucleophilic heteroaromatic substitution reaction using a trimethylammonium triflate precursor (**1**) and the [^18^F]F^−^/K_2.2.2._/K_2_CO_3_ fluorination system according to the manual procedure described by Inkster et al. [17] (Scheme 3). The complete radiosynthesis was performed with an automated synthesis module. The setup of the module is described in the Materials and Methods section. Briefly, after trapping and elution of [^18^F]fluoride from an anion exchange cartridge, the labeling reaction was performed in DMSO at 120 °C. For isolation of **[^18^F]2**, the crude reaction mixture was diluted with water/acetonitrile and then directly applied to the implemented semi-preparative HPLC system (for the chromatogram, see Figure S2 in Supplementary Materials). For the subsequent final purification by solid-phase extraction (SPE), the radiotracer fraction was collected in the collecting vial preloaded with water and a small amount of 1M NaOH to neutralize the acidic eluent containing 0.05% trifluoro acetic acid before loading on a C18 light cartridge. In contrast to the low sorption efficiencies (27–42%) described by Inkster et al. when using a light cartridge, with this procedure, 87 ± 1% (n = 5) of activity could be loaded. The Inkster group improved the SPE step by using two “full-size” C18 plus cartridges followed by elution of the activity with methanol and subsequent evaporation of the solvent. This process caused slight activity losses during evaporation and resulted in considerable longer total synthesis times (103 min) but reasonable radiochemical yields of 39 ± 9% could be achieved [17]. Nevertheless, this approach was less suitable for our purpose, because we aimed to generate the prosthetic group in a shorter time. In the next step, the activity loaded cartridge was removed from the synthesis module and **[^18^F]2** was manually eluted with a small volume of DMSO (300–400 µL) ready for subsequent click reactions. With this procedure, only 5 ± 1% (n = 8) of the activity remained on the cartridge resulting in total radiochemical yields (RCYs) of 38 ± 5 % (n = 10) and radiochemical purities of ≥99%. Thus, the results are comparable to the ones reported by Inkster et al. [17]. However, the entire process was reduced to about 60 min. 

#### 3.3.2. Radiosynthesis of the Teroligomer **[^18^F]fluoro-o14PEGMA** by Conjugation of **o14PEGMA-N_3_** with **[^18^F]2**

In order to find most suitable CuAAC reaction conditions for our purpose, an initial screening of different reaction parameters was performed using **[^18^F]2** and azido-TEG-amine as an easily available and broadly soluble coupling reagent (for details and Scheme S1, see Supplementary Materials). According to the literature, CuSO_4_ and sodium ascorbate (NaAsc) as the reducing agent and a mixture of water/DMSO (4:1 (*v*/*v*)) as solvent were used. In a set of experiments, the following parameters were investigated: (i) the temperature (40, 70 and 90 °C), (ii) the concentration of the azide, (iii) the reaction time (5–60 min.) and (iv) the ratio of azide to CuSO_4_. The molar ratio of CuSO_4_ to NaAsc was kept constant at 1 to 4 to ensure effective reduction of Cu(II) to Cu(I). The formation of the radioactive triazole coupling product was monitored using radio-HPLC at different time points (for data, see Figure S3 in Supplementary Materials). In brief, the selected reaction system was shown to be suitable, and the formation of the coupling product could already be observed after short reaction times of 5 to 15 min. The conversion was mainly depending on the concentration of the azide and the reaction temperature. High yields and short reaction times could be achieved with high azide concentrations (25 µmol) already at low temperature (40 °C). However, the azide concentration is a parameter that is not very variable if the reaction conditions have to be transferred to the intended coupling reaction with the polymer, since the azide functionalities in the polymer only represent a small fraction of the molecular structure. Therefore, higher temperatures revealed to be necessary to achieve reasonable conversion yields for these reaction partners. 

Based on the initial screening results, the first CuAAC reactions between the azide functionalized teroligomer **o14PEGMA-N_3_** and **[^18^F]2** (Scheme 3) were performed at 90 °C using (i) different polymer concentrations, (ii) a polymer to CuSO_4_ ratio of 1:2 and (iii) water/DMSO as the solvent mixture (4:1 (*v*/*v*), 500 µL). The reaction mixtures were analyzed by radio-SEC and radio-RP-HPLC, taking samples at different time points. During the optimization of the reaction conditions, again a strong variation of the yields was observed depending on the amount of teroligomer. For example, using 2 mg of **o14PEGMA-N_3_** resulted in RCYs of about 60%, whereas 4 mg yielded approximately 80%. Moreover, it was found that already after 2–3 min of reaction time, the radioactive alkyne partner was quantitatively consumed and the coupling product formed. Longer reaction times lead to the decomposition of the radiolabeled product. In contrast to the initial screening with azido-TEG-amine as azide component, the ratio of polymer to CuSO_4_ played an important role. A minimum molar ratio of 1:20 was necessary to achieve satisfying conversion yields (for comparison, the ratio azido-TEG-amine to CuSO_4_ was 1:2). This excess of copper salt was probably needed due to a partial complexation of Cu ions by the carboxylate moieties of **o14PEGMA-N_3_**. 

**Figure 3:**
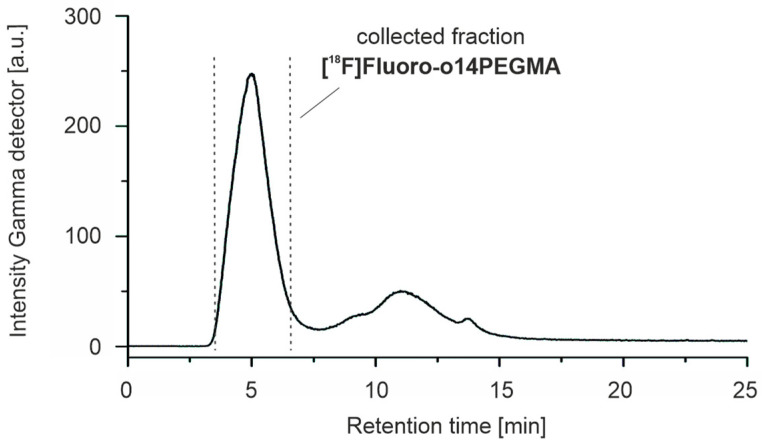
Exemplary radio-chromatogram of the semi-preparative SEC purification of the radiolabeled teroligomer **[^18^F]fluoro-o14PEGMA** (conditions: 2 HiTrap^TM^ desalting 5 mL columns in line, 3.75 mM aq. phosphate pH 7, flow 1.0 mL/min).

For the purification of the radiolabeled teroligomer **[^18^F]fluoro-o14PEGMA**, solid-phase extraction and subsequent isolation by size-exclusion chromatography was performed. The SPE step was found to be necessary to remove the excess of Cu ions. For this purpose, Sep-Pak^®^ C18 and CHROMAFIX SB (anionic exchanger) cartridges of different size were tested. The best sorption efficiencies of the radiolabeled polymer could be obtained with the use of the CHROMAFIX SB cartridge, which correlates well with the anionic character of this amphiphilic polymer. The elution was tested under basic (100 mM PO_4_^3−^, 1.0 M NaOH) and acidic (0.1 and 1.0 M HCl) conditions, of which aqueous 1.0 M HCl was found to be most suitable as mainly the desired polymer was eluted with sufficient recovery. For final purification, the acidic eluate was neutralized and manually subjected to the SEC setup consisting of two in-line connected HiTrap^TM^ columns with aqueous phosphate buffer as eluent (chromatogram see Figure 3). The final product was obtained by the collection of the radiolabeled polymer fraction without the need for further manipulation. All manual steps of the polymer radiolabeling and purification process occurred over about 40 min, and an RCY of about 15% (n = 3) was achieved (calculated on the basis of applied **[^18^F]2**). A rather high loss of activity has been observed during the SEC purification, with almost 30% of activity remaining on the columns. Overall, formulation of the pure **[^18^F]fluoro-o14PEGMA** could be obtained with a total synthesis time of about 120 min.

**Figure 4:**
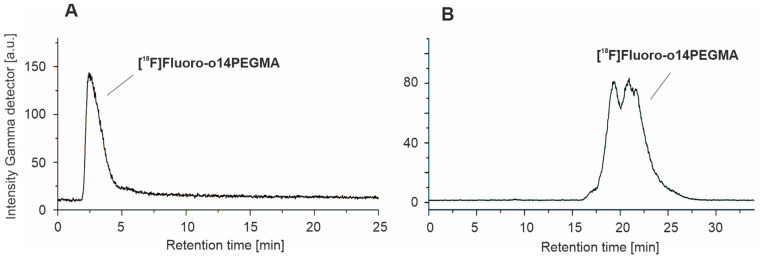
Exemplary radio-chromatograms of the analytical quality control of **[^18^F]fluoro-o14PEGMA** using (**A**) radio-SEC (HiTrap^TM^ desalting 5 mL column, 10% ethanol/25 mM aq. phosphate pH 7, flow 1.0) and (**B**) radio-RP-HPLC (Reprosil Pur C18 AQ 250 × 4.6 mm, gradient ACN/20 mM aq. NH_4_OAc, flow 1.0).

The analyses of the radiolabeled teroligomer **[^18^F]fluoro-o14PEGMA** fraction was performed using radio-SEC and radio-RP-HPLC. Figure 4A,B shows representative examples of the corresponding analytical radio-chromatograms obtained with the purified ^18^F-labeled polymer. As expected, **[^18^F]fluoro-o14PEGMA** eluted in the SEC system at short retention times close to the void volume due to its macromolecular structure (Figure 4A). A rather pronounced tailing was observed over the entire elution process indicating undesired adsorption processes of **[^18^F]fluoro-o14PEGMA** on the phase material, a phenomenon which is well known [38]. This observation in the analytical scale corresponds to the observed activity loss on the HiTrap^TM^ columns during semi-preparative SEC purification. In the RP system with gradient mode (Figure 4B), adsorption is the main determinant for retention. As a consequence, the terpolymer does not elute before a certain concentration of the organic modifier is reached during the gradient run, which allows the desorption [38]. 

## 4. Conclusions

This study presents a procedure for the generation and analysis of modified amphiphilic polymers covalently labeled with fluorine-18. Applying a copper-catalyzed click-type cycloaddition reaction, the rather hydrophilic heteroaromatic prosthetic group **[^18^F]2** was coupled with an azide-functionalized teroligomer within 2–3 min. The partially anionic character of the radiolabeled product **[^18^F]fluoro-o14PEGMA** allowed for its prepurification via SPE on an anion-exchange cartridge to remove the bulk of reaction additives. Final purification was performed by size-exclusion chromatography, and the radiolabeled teroligomer could be obtained as a phosphate-buffered solution that is ready for further use. The suitability of this new radiofluorinated polymer formulation for the generation of radiolabeled CaP-NPs is subject of current investigation.

## Data Availability

All data are contained within the article and Supplementary Materials.

## Supplementary Materials

The following supporting information can be downloaded at: https://www.mdpi.com/article/10.3390/nano13142095/s1, Figure S1: ^1^H NMR spectra of **1** and **2**; Figure S2: Semi-preparative HPLC of **[^18^F]2**; Figure S3: Formation of the triazole coupling product **[^18^F]3,** which is dependent on reaction time, temperature and concentration; Scheme S1: Click-coupling reaction of **[^18^F]2** with azido-TEG-amine.
