# Supplementary material for: Radiofluorination of an Anionic, Azide-Functionalized Teroligomer by Copper-Catalyzed Azide-Alkyne Cycloaddition
Source: Nanomaterials (Basel). 2023 Jul 18;13(14):2095. doi: 10.3390/nano13142095 (PMC10385230; doi:10.3390/nano13142095)
Supplement: Supplementary file 1 [file nanomaterials-13-02095-s001.zip › nanomaterials-2427319-supplementary.pdf]

nanomaterials

MDPI

Supplementary Materials

Radiofluorination of an Anionic, Azide-Functionalized Teroligomer by Copper-Catalyzed Azide-Alkyne Cycloaddition

Barbara Wenzel [1,*], Maximilian Schmid [2,3], Rodrigo Teodoro [1], Rareş-Petru Moldovan [1], Thu Hang Lai [1], Franziska Mitrach [2], Klaus Kopka [1,4], Björn Fischer [3], Michaela Schulz-Siegmund [2], Peter Brust [1] and Michael C. Hacker [2,3,*]

[1] Department of Neuroradiopharmaceuticals, Institute of Radiopharmaceutical Cancer Research, Helmholtz-Zentrum Dresden-Rossendorf, Leipzig, 04318, Germany; r.teodoro@life-mi.com (R.T.); r.moldovan@hzdr.de (R.-P.M.); t.lai@hzdr.de (T.H.L.); k.kopka@hzdr.de (K.K.); peterbrustdeu@aol.com (P.B.)

[2] Institute of Pharmacy, Pharmaceutical Technology, Leipzig University, Leipzig, 04317, Germany; maximilian.schmid@uni-leipzig.de (M.S.); franziska.mitrach@uni-leipzig.de (F.M.); schulz@uni-leipzig.de (M.S.-S.)

[3] Institute of Pharmaceutics and Biopharmaceutics, Heinrich Heine University Düsseldorf, Düsseldorf, 40225, Germany; bjoern.fischer@hhu.de

[4] Faculty of Chemistry and Food Chemistry, School of Science, Technical University Dresden, Dresden, 01069, Germany

* Correspondence: b.wenzel@hzdr.de (B.W.); mch@mchlab.de (M.C.H.)

1)1. H NMR spectra of 1 and 2

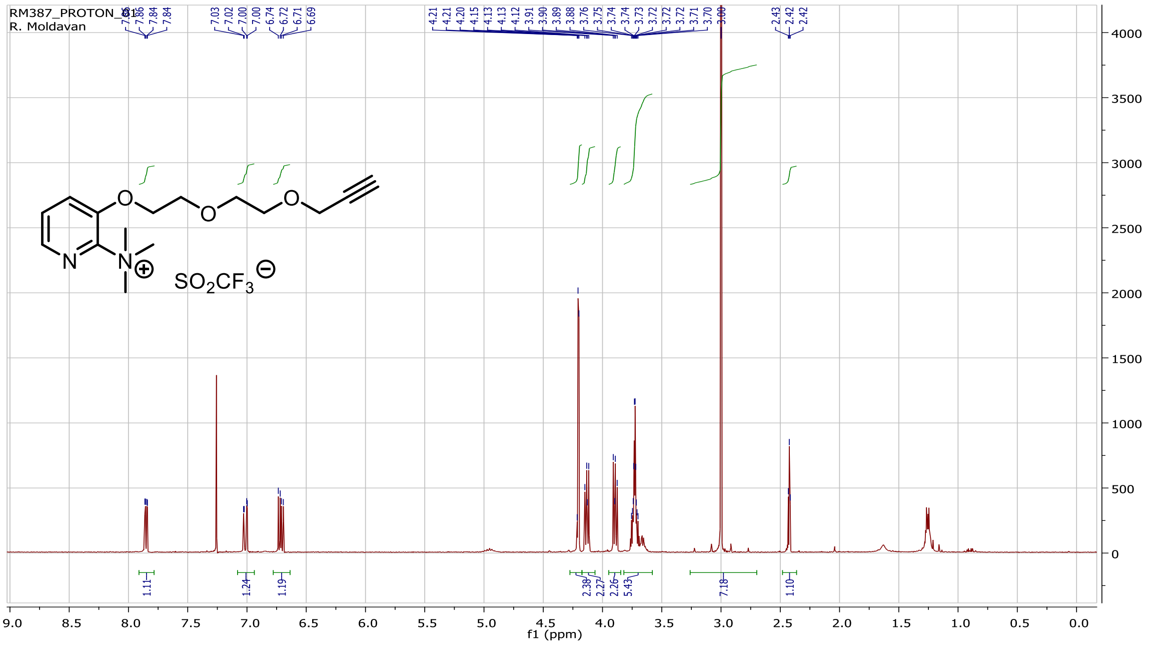

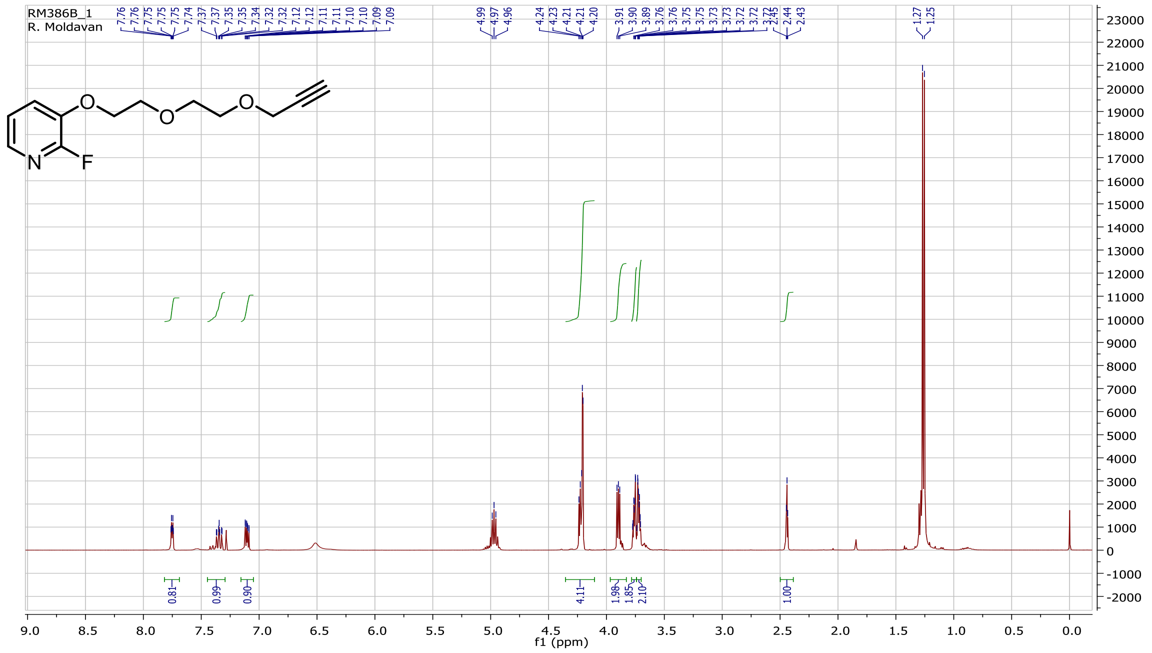

Figure S1. ^{1}H NMR spectra of the precursor **1** and the reference compound **2.**

2). Semi-preparative HPLC of [^{18}F]2

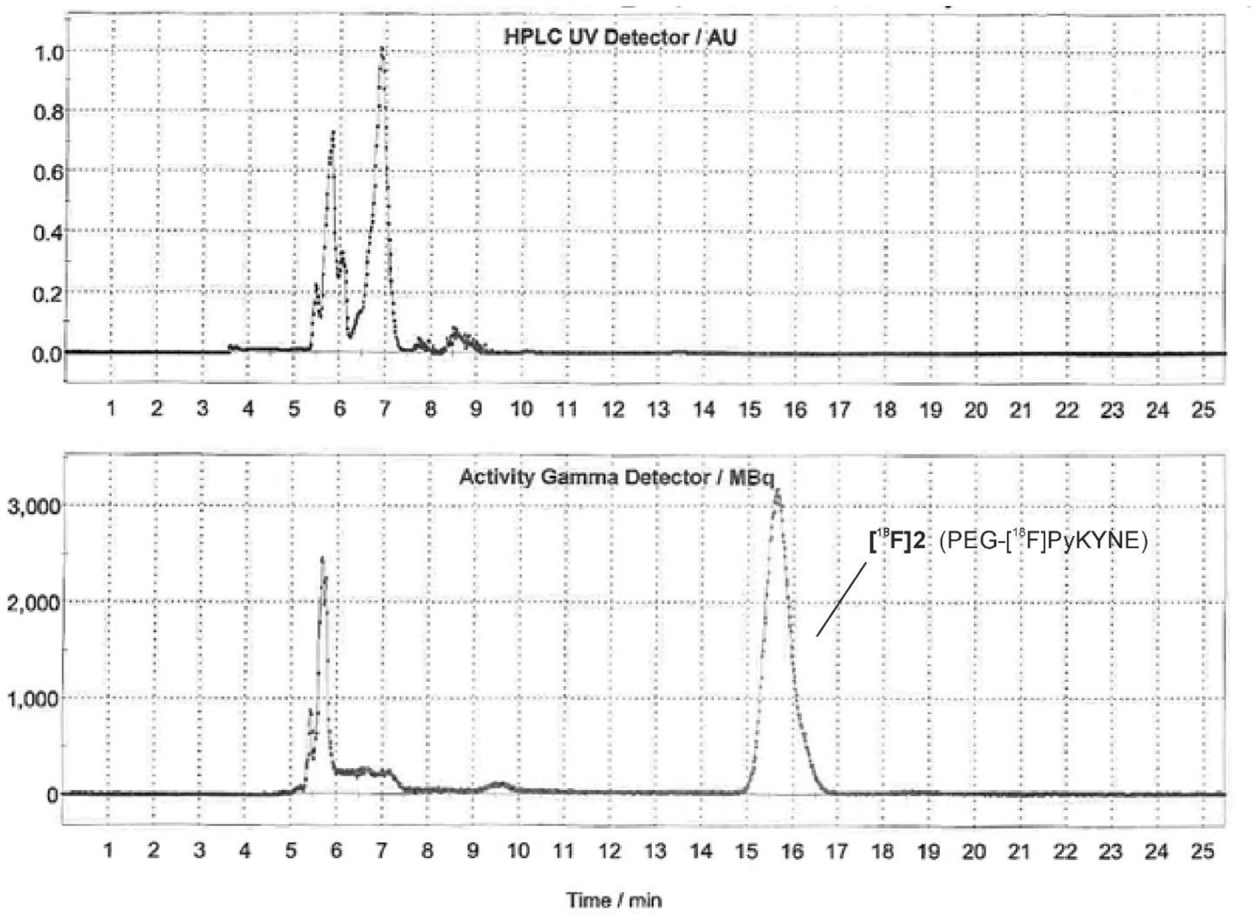

Figure S2. Representative chromatogram of the semi-preparative separation of **[^{18}F]2** during the automated radiosynthesis. Conditions: Reprosil Pur C18 AQ (250 × 10 mm), 35% ACN/H_2O/0.05% TFA, Flow 4.0 mL/min.

3). Screening of click coupling reaction parameters

In order to find most suitable CuAAC reaction conditions, a screening of different reaction parameters was performed using **[18F]2** and azido-TEG-amine (2-(2-(2-(2-azidoethoxy)ethoxy)ethoxy)ethan-1-amine) as easy available coupling reagent (Scheme S1). A typical coupling reaction was performed as follows (the quantities of reactants were adjusted according to the respective setup): The azido-TEG-amine (5 µl, 25 µmol) was dissolved in 100 µL water and mixed with freshly prepared aqueous 0.5 M sodium ascorbate (300 µl, 150 µmol] and 100 µL (150 - 350 MBq) of **[18F]2** in DMSO. Thereafter, aqueous 0.5 M $CuSO_4$ solution (100 µL, 50 µmol) was added under stirring and the reaction mixture was heated up to the respective temperature. Samples were taken after 5, 15, 30 and 60 minutes. The reaction was performed under argon atmosphere and all solvents were saturated with argon before usage. The general total reaction volume was 500 µL and achieved by adding the respective needed amount of water.

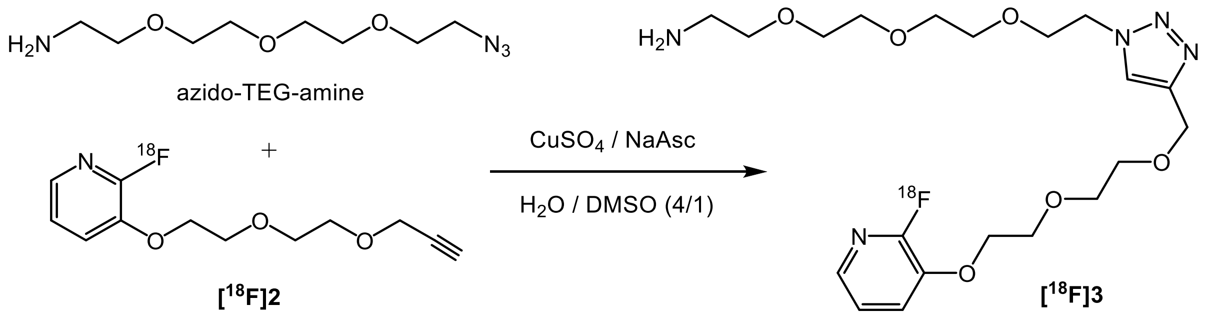

Scheme S1. Click coupling reaction of **[18F]2** with azido-TEG-amine for reaction parameter screening.

To determine the radiochemical yield of the formation of the triazole **[18F]3**, radio-HPLC was used under following conditions: Reprosil-Pur C18-AQ column (250 x 4.6 mm; 5 µm; Dr. Maisch HPLC GmbH; Germany) with ACN/aq. 20 mM NH_4OAc (pH 6.8) as eluent mixture and a flow of 1.0 mL/min (gradient: eluent A 10% ACN/aq. 20 mM NH_4OAc; eluent B 90% ACN/aq. 20 mM NH_4OAc; 0–10 min 100% A, 10–25 min up to 100% B, 25–30 min 100% B, 30–31 min up to 100% A, 31–35 min 100% A.

The identity of **[18F]3** was confirmed by comparison of the retention time with the corresponding non-radioactive reference compound **3** (2-(2-(2-(2-(4-((2-(2-((2-fluoropyridin-3-yl)oxy)ethoxy)ethoxy)methyl)-1H-1,2,3-triazol-1-yl)ethoxy)ethoxy)ethoxy)ethan-1-amine), which was synthesized accordingly in analytical scale. The obtained triazole **3** was verified by LC-MS (Dionex Ultimate 300 with LPG-3400SD pump, WPS-3000 TSL autosampler, DAD-3000 diode array detector coupled with a low-resolution mass spectrometer MSQ 3000 (Thermo Fisher Scientific Inc., Waltham, MA, USA) using a Reprosil-Pur Basic HD column (150 x 3.0 mm; 3 µm; Dr. Maisch HPLC GmbH; Germany) with ACN/aq. 20 mM NH_4OAc (pH 6.8) as eluent mixture and a flow of 0.6 mL/min (gradient: eluent A 10% ACN/aq. 20 mM NH_4OAc; eluent B 90% ACN/aq. 20 mM NH_4OAc; 0–2 min 100% A, 2–15 min up to 100% B, 14–17 min 100% B, 17–18 min up to 100% A, 18–20 min 100% A. LR-MS m/z calculated for $C_{20}H_{32}FN_5O_6$ 457.23; found 458 $[M+H^+]^+$ and 520 $[M+CH_3CN+Na^+]^+$.

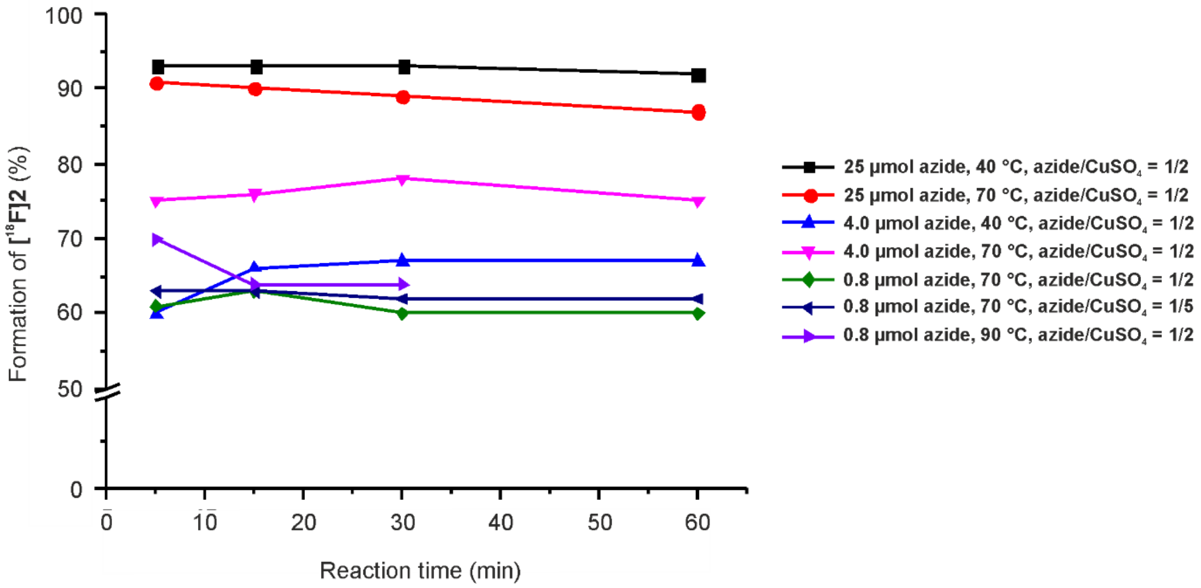

Figure S3. Formation of the triazole coupling product **[^{18}F]3** in dependence on reaction time, temperature and concentration.
